# Mandibular Bone Resorption Following Chin Augmentation: A Systematic Review

**DOI:** 10.3389/fsurg.2022.815106

**Published:** 2022-03-25

**Authors:** Andy Wai Kan Yeung, Natalie Sui Miu Wong

**Affiliations:** ^1^Oral and Maxillofacial Radiology, Applied Oral Sciences and Community Dental Care, Faculty of Dentistry, The University of Hong Kong, Pok Fu Lam, Hong Kong SAR, China; ^2^Oral and Maxillofacial Surgery, Faculty of Dentistry, The University of Hong Kong, Pok Fu Lam, Hong Kong SAR, China

**Keywords:** craniofacial surgery, radiology, cone-beam computed tomography, chin, diagnosis

## Abstract

**Background:**

Chin implants have a long history, and its usage may be associated with mandibular bone resorption.

**Objectives:**

This report analyzed data on this topic from existing literature to evaluate the overall resorption rate and scientific impact in terms of citations.

**Method:**

PubMed, Web of Science, Scopus, and Google Scholar databases were searched to identify relevant publications. The search string was as follows: (chin) AND (augment^*^ OR implant^*^) AND (resorb^*^ OR resorp^*^) AND (bone OR osseous). A study was eligible if it recruited human subjects and reported resorption following any chin implantation based on radiographic examination.

**Results:**

Twenty-eight patient studies were identified. Publication year seemed to have no effect on the mean depth of bone resorption and its prevalence as reported by the studies. The increased mean number of follow-up years seemed to have no effect on its prevalence but seem to be associated with deeper bone resorption. The majority of the studies had <5 years of follow-up and reported a mean of <2 mm of bone resorption. The most cited study had 69 citations. Citations rarely came from radiology journals. A limitation was that unpublished data could not be analyzed.

**Conclusions:**

Mandibular bone resorption caused by chin implants of various materials is a common phenomenon. Its recognition and studies with a longer follow-up period should be further promoted.

## Introduction

**Figure 1:**
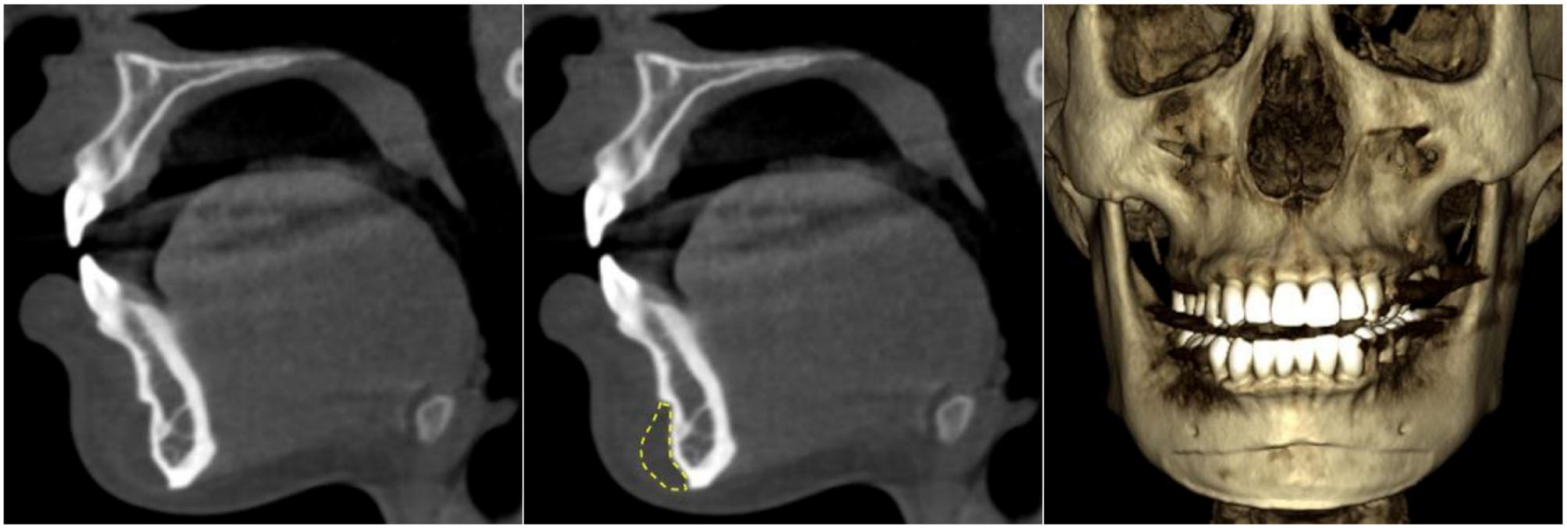
Mandibular bone resorption on the labial surface caused by a chin implant observed with cone-beam CT. The resorption was obvious in both sagittal view and volume rendering. The chin implant is circled yellow.

Chin implants have a long history. The earliest reports of causing bone resorption at the augmentation site could be traced back to the 1960s (1). The extent of bone resorption could be evaluated by plain radiography, such as panoramic imaging (to evaluate from the mesiodistal dimension, or “width”) and lateral cephalography (to evaluate the buccolingual extent, or “depth”). Three-dimensional imaging, such as cone-beam computed tomography (CBCT), would give a better visualization of bone resorption. The resorption might be influenced by the positioning of the chin implant. In addition, it is usually more severe at the superior end due to the contraction force of the mentalis muscle (2). Figure 1 shows a typical appearance of mandibular bone resorption caused by a chin implant, leaving an imprint on the labial surface of the mandible. If the resorption extended to the periapical region of the mandibular anterior teeth, the neurovascular bundles supplying these teeth could be severed, rendering them non-vital.

Half a century has elapsed since the earliest reports on the bone resorption caused by chin augmentation. This study aimed to evaluate the scientific evidence on radiographic bone resorption following chin implantation. The objectives were to analyze the reported prevalence and depth of such bone resorption and to reveal the amount of scientific attention paid to this topic in terms of citation count. To view the citation performance from another perspective, the relative citation ratio (RCR), a metric that indicates the relative citation performance of a publication as compared to other publications in its research area (with >1 means above the average) (3, 4), was recorded.

## Materials and Methods

**Figure 2:**
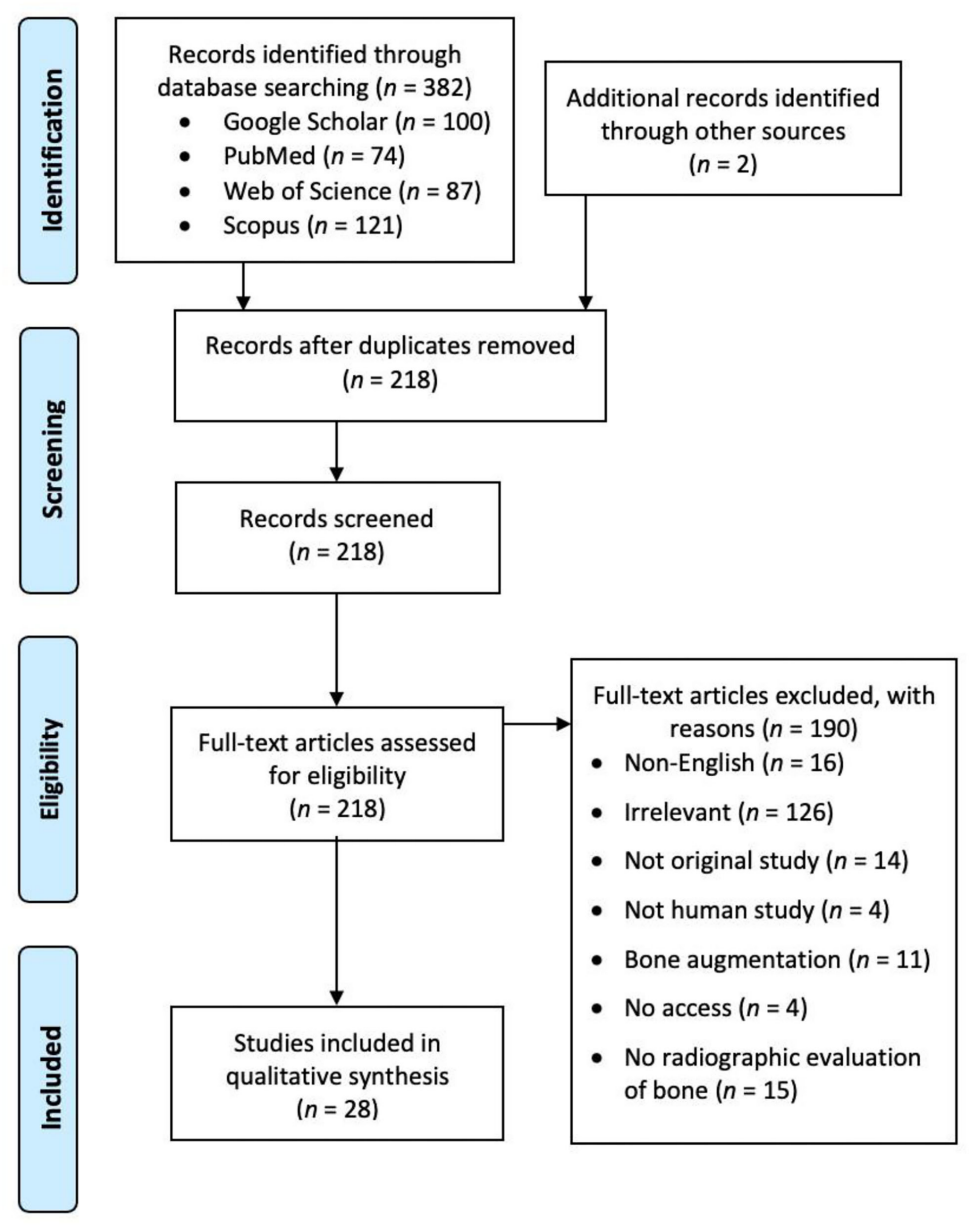
A preferred reporting items for systematic reviews and meta-analyses (PRISMA) flow chart showing the screening process of the literature search.

On June 17, 2021, four literature databases (PubMed, Web of Science, Scopus, and Google Scholar) were searched. The search string was as follows: (chin) AND (augment^*^ OR implant^*^) AND (resorb^*^ OR resorp^*^) AND (bone OR osseous). For PubMed, the search covered the fields of article title and abstract. For Web of Science and Scopus, the search covered article title, abstract, and keywords. Because Google Scholar searched all fields of the publications including their full texts and reference lists, the abovementioned search string returned with many irrelevant articles focusing on dental implants. To be more specific, the search string was modified for Google Scholar as “chin augmentation resorption,” and the first 100 publications were screened. Hand search on the reference lists of the screened publications was also performed. No limitation was set for the publication year, but the publications must be written in English. The searches initially returned with 384 publications. After removing duplicates, 218 publications remained. The statement of questions being addressed with reference to participants, interventions, comparisons, outcomes, and study design (PICOS) was as follows: participants—human subjects; interventions—chin implantation but not bone augmentation; comparisons—within-subject comparison; outcome—radiographic evaluation of mandibular bone resorption; and study design—a longitudinal study. After screening and excluding unsuitable publications for specific reasons, 28 studies remained for review (Figure 2). Ethical approval was not applicable to this review. This systematic review was not pre-registered in PROSPERO or other databases.

## Results

**Figure 3:**
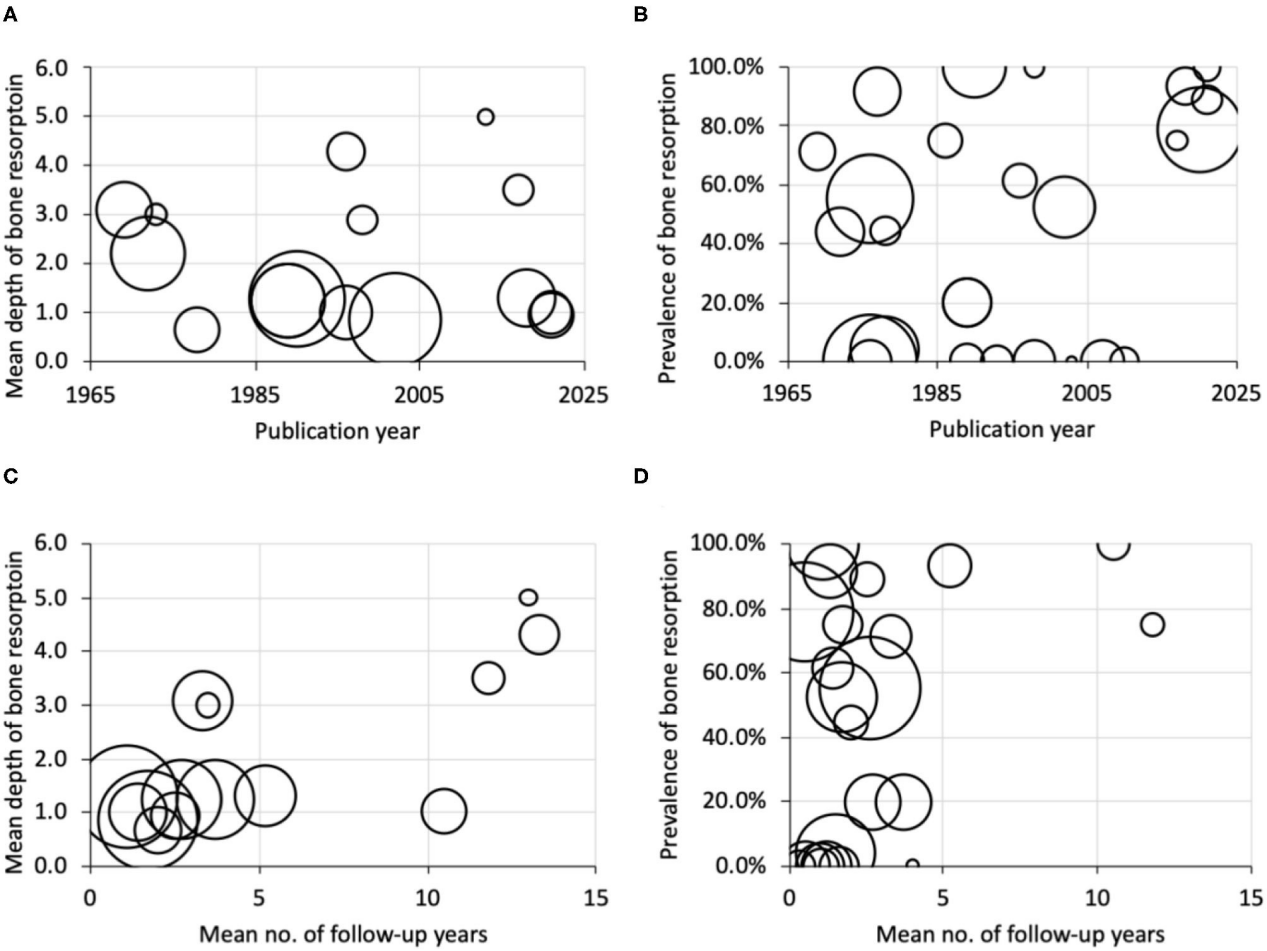
Bubble plots of publication year against **(A)** mean depth of bone resorption, **(B)** prevalence of bone resorption, and of the mean number of follow-up years against, **(C)** mean depth of bone resorption, and **(D)** prevalence of bone resorption. Bubble size indicates the sample size.

The 28 identified studies are listed in Table 1 (1, 2, 5–30). Several key points to summarize the data were as follows:

(1) Publication year seemed to have no effect on the mean depth of bone resorption and its prevalence as reported by the studies (Figures 3A,B).
(2) Increased mean number of follow-up years seemed to associate with deeper bone resorption. However, readers should notice that the majority of the studies had <5 years of follow-up and reported a mean of <2 mm of bone resorption. Only four studies had >10 years of follow-up on average, with one of them reporting a resorption rate of 75%, whereas the remaining three reported positive cases only (Figure 3C).
(3) Mean number of follow-up years seemed to have no effect on the prevalence of bone resorption as reported by the studies (Figure 3D). Seven studies reported 0% prevalence. On the other hand, 5 studies reported a prevalence of >85%.
(4) Sub-periosteal placement of chin implants seemed to be the mainstream. Only two studies solely evaluated supra-periosteal chin implants. Both reported an absence of bone resorption.
(5) Silicone (silastic) was the most common implant material, with highly varied depth and prevalence of bone resorption.
(6) These reports were mainly published in surgery journals. Only one was published in a radiology journal (neuroradiology).

**Table 1:** Details of the 28 included studies of reporting mandibular bone resorption caused by chin implant.

**References**	**No. of patients**	**Gender**	**Age, mean (range)**	**Bone** **resorption** **prevalent rate**	**Mean radiographic bone resorption depth of +ve cases (mm)**	**Mean (range) of follow-up** **years**	**Material for chin implant**	**Supra-/sub-periosteal implant**	**Total citations (citations by Radiology journals)**	**Relative citation ratio (RCR)**
Robinson (1)	14	5M 9F	29.2 (14–60)	71.4%	3.1	3.3 (1–6)	Silicone; acrylic	Sub	69 (5)	NA
Robinson (5)	25	?	?	44.0%	2.2	?	Silicone; acrylic	?	44 (0)	NA
Jobe et al. (6)	2	1F 1?	?	(Positive cases only)	3	3.5 (1.5–5.5)	Silicone	?	49 (2)	NA
Beekhuis (7)	100	?	?	0%		?	Polyamide mesh	Supra	17 (0)	NA
Parkes et al. (9)	19	?	?	0.0%		All at 0.5	Proplast I	Supra	24 (0)	NA
Friedland et al. (8)	85	14M 71F	(21–45)	55.3%	(13–27% of total thickness of the mandibular symphysis)	(0.1–5)	Silicone	Both	58 (0)	NA
Dann and Epker (10)	24	?	19.2	91.7%	?	1.3 (0.3–2.3)	Proplast I	Sub	38 (2)	NA
Snyder et al. (12)	50	?	?	4.0%	?	(1–2)	Silicone	Both	10 (0)	NA
Feuerstein (11)	9	?	?	44.4%	0.67	(1–3)	Silicone	Supra- for central part; sub- for lateral parts	11 (0)	NA
Peled et al. (13)	12	?	?	75.0%	?	(1.5–2)	Polydimethyl siloxane	Both	27 (2)	4.39
Moenning and Wolford (14)	25	7M 18F	24.3 (12–50)	20.0%	1.25	3.7 (2–6.8)	Proplast I	Sub	15 (0)	1.46
	25	12M 13F	24.4 (13–39)	20.0%	1.24	2.7 (2.1–4.3)	Proplast II	Sub		
	12	4M 8F	27.2 (13–54)	0.0%		1.6 (1–2.5)	Porous block of hydroxyapatite	Sub		
Guyuron and Raszewski (15)	42	?	(13–64)	100%	1.3	All at 1.1	Proplast II	Sub	46 (0)	4.26
Holmström et al. (16)	10	?	23 (17–33)	0.0%		All at 1	PMMA + pHEMA (HTR, Biomet Inc, USA)	Sub	5 (0)	0.46
Vuyk (17)	13	?	?	61.5%	1	1.4 (0.1–3.8)	Silicone	Sub	15 (0)	0.73
Matarasso et al. (2)	6	6F	37.3 (22–62)	(Positive cases only)	4.3	13.3 (4–30)	Silicone	?	34 (1)	2.56
Karras and Wolford (19)	18	3M 15F	26.3 (14–44)	0%		At least 1	PMMA + pHEMA (HTR, Biomet Inc., USA)	Sub	15 (0)	0.88
Abrahams and Caceres (18)	4	?	?	100.0%	2.9	?	Silicone	?	7 (3)	0.46
Saleh et al. (20)	40	10M 30F	29.1 (16–50)	52.5%	0.86	1.7 (0.7–5)	Silicone	Sub	8 (0)	0.33
Viterbo (21)	1	?	43	0%		4	Conchal cartilage	Sub	9 (0)	0.45
Gürlek et al. (22)	20	8M 12F	(18–39)	0%		1.2 (0.7–1.7)	Polyethylene	Sub	14 (0)	0.66
Mohammad et al. (23)	8	?	(15–35)	0.0%		All at 0.3	Polyethylene (Medpore)	?	4 (0)	0.37
Shi et al. (24)	1	1F	35	(Positive cases only)	5	13	ePTFE	?	6 (0)	0.5
Polo (25)	4	1M 3F	34 (32–39)	75.0%	3.5	11.8 (10–17)	Silicone; mersilene mesh	?	5 (0)	0.58
Sciaraffia et al. (27)	15	?	34 (14–57)	93.3%	1.3	5.2 (1–17)	Silicone	Sub	3 (0)	0.94
Guo et al. (26)	1	1F	31	(Positive cases only)	?	?	Hyaluronic acid	?	2 (0)	0.25
Yamazaki et al. (28)	1	1F	39	(Positive cases only)	(Approaching lingual cortex)	14	Silicone	?	0 (0)	NA
Guo et al. (29)	80	3M 77F	25.9 (19–36)	78.8%	?	All at 0.5	Hyaluronic acid	?	3 (0)	2.44
Ortiz-Díaz et al. (30)	9	9F	47.6	88.9%	0.94	(1–5)	Silicone	?	0 (0)	NA
	8	8F	47.6	100%	1.02	(6–15)	Silicone	?		

*No. of citations was according to Web of Science electronic platform. Relative citation ratio (RCR) was from dimensions electronic platform. NA, not available*.

Data from Web of Science indicated that the most cited paper was Robinson (1) with 69 citations. It was also the paper with the highest citations from dental or medical radiology journals (5 citations). Only six of the 28 studies (21.4%) received citations from radiology journals. Overall, the 28 studies had a mean ± SD of 19.2 ± 19.1 citations, with 0.5 ± 1.2 citations from radiology journals.

## Discussion

This work identified 28 studies reporting the prevalence and mean depth of mandibular bone resorption caused by chin implants in patients. They reported a very wide range of prevalence, specifically from 0 to 100%. The increased mean number of follow-up years seemed to associate with deeper bone resorption, but most studies had <5 years of follow-up and reported a mean of <2 mm of bone resorption. Silicone was the most common chin implant material used, and sub-periosteal placement was the most common technique. Subjects were mostly females who were young adults or middle-aged. The implant material, placement technique, and gender did not seem to have a profound effect on the resorption rate.

Unlike some incidental radiographic findings, such as medial sigmoid depression in the mandible for which the reports were seldom cited (31), the mandibular bone resorption caused by chin implant is well known and the reports were frequently cited. This review showed that these reports were cited 19.2 times on average, a figure even higher than that of osteoporosis detection by panoramic imaging (32). Surprisingly, this phenomenon was rarely mentioned by radiology journals and, thus, received few citations by the latter. In terms of RCR, most of the studies had a score <1, meaning that their citation performance was still below the average compared to other papers in the radiology and plastic surgery research areas. Chin implant and its associated bone resorption have important clinical implications as they may affect the mental foramen, causing chin hypoesthesia/dysesthesia and tooth root damage together with other potential complications (33). Evaluation of the positioning of a chin implant or the extent of bone resorption heavily relies on radiographic examinations that are not intrusive or traumatic. With the increasing number of patients undergoing plastic surgery in the head and neck region including the chin, a recommendation should be made to promote the awareness of this mandibular bone resorption. More long-term longitudinal research should be conducted on this topic to better understand the temporal profile of the progression of this bone resorption, e.g., whether it would progress continuously with time or become stable after a certain period. This bone resorption phenomenon is probably better known by oral and maxillofacial surgeons and plastic surgeons, but not dentomaxillofacial radiologists or general dentists. Regardless, they should all be more aware of bone resorption and its associated consequences. Readers should be aware that one limitation of this study was that unpublished data could not be analyzed. In addition, a citation could be supporting the cited findings, mentioning them, or contrasting them, which was not evaluated in this report.

## Conclusions

To conclude, mandibular bone resorption caused by chin implants of various materials is frequently documented. It is an uncommon yet highly important post-surgery complication as the resorption may eventually erode the mental foramen and the roots of mandibular teeth. Hence, its recognition should be promoted. In addition, most studies had <5 years of follow-up with a mean of <2 mm of bone resorption. Studies with >10 years of follow-up had very small sample sizes. When all studies were considered together, an increased number of follow-up years seemed to associate with deeper bone resorption. Hence, longer follow-up periods in future studies should be further promoted.

## Data Availability Statement

The original contributions presented in the study are included in the article/supplementary material, further inquiries can be directed to the corresponding author/s.

## Author Contributions

AY is responsible for design, data collection, analysis, and manuscript writing. NW is responsible for data collection and manuscript writing. Both authors contributed to the article and approved the submitted version.

## Funding

This work was supported by departmental funding.

## Conflict of Interest

The authors declare that the research was conducted in the absence of any commercial or financial relationships that could be construed as a potential conflict of interest.

## Publisher's Note

All claims expressed in this article are solely those of the authors and do not necessarily represent those of their affiliated organizations, or those of the publisher, the editors and the reviewers. Any product that may be evaluated in this article, or claim that may be made by its manufacturer, is not guaranteed or endorsed by the publisher.
